# Dihydropyrimidinase‐like 2 can serve as a novel therapeutic target and prognostic biomarker in acute myeloid leukemia

**DOI:** 10.1002/cam4.5531

**Published:** 2023-01-09

**Authors:** Fenglin Li, Qing Ling, Jiaying Lian, Ying Chen, Chao Hu, Min Yang, Xiang Zhang, Chenying Li, Shihui Mao, Wenle Ye, Xia Li, Xiangjie Lin, Wenwen Wei, Xin Huang, Jiajia Pan, Yu Qian, Jinghan Wang, Ying Lu, Jie Jin

**Affiliations:** ^1^ Department of Hematology, the First Affiliated Hospital Zhejiang University, College of Medicine Hangzhou Zhejiang People's Republic of China; ^2^ Department of Hematology The affiliated people's hospital of Ningbo University Ningbo China; ^3^ Zhejiang Provincial Key Lab of Hematopoietic Malignancy Zhejiang University Hangzhou Zhejiang People's Republic of China; ^4^ Zhejiang University Cancer Center Hangzhou Zhejiang China

**Keywords:** AML, biomarker, DPYSL2, HHT, PI3K/Akt, prognostic

## Abstract

**Background:**

Identifying therapeutic targets and prognostic biomarkers significantly contributes to individualized treatment of acute myeloid leukemia (AML). Dihydropyrimidinase‐like 2 (DPYSL2) expression was decreased in homoharringtonine (HHT)‐resistant AML cells, which were established by our group. DPYSL2 plays an important role in axon growth and has oncogene effect in glioblastoma. However, little research has been conducted to investigate the function of DPYSL2 in AML pathogenesis.

**Methods:**

Auto‐docking was used to reveal the targeting relationship between HHT and DPYSL2. Overall survival (OS), event‐free survival (EFS), and relapse‐free survival (RFS) were used to evaluate the prognostic impact of DPYSL2 for AML. ShRNA was used to knockdown the expression of SPATS2L. Apoptosis was assessed by flow cytometry. In vivo growth and survival were assessed using a xenotransplantation mice model. RNA sequencing was performed to elucidate the molecular mechanisms underlying the role of SPATS2L in AML and were confirmed by Western blot.

**Results:**

We found DPYSL2 was the target of HHT. Next, we found AML cell lines and patients had higher DPYSL2 expression levels than the normal samples. Further multivariate analysis demonstrated that high DPYSL2 expression was an independent poor prognostic factor for OS, EFS, and RFS in AML. Inhibition of DPYSL2 expression suppressed cell growth, induced apoptosis in AML cell lines, and prolonged the survival of AML xenograft NCG mice. Through RNA‐seq analysis from TCGA and our data, the JAK2/STAT3/STAT5‐PI3K P85/AKT/GSK3b axis was thought to be the critical pathway in regulating DPYSL2 in AML development.

**Conclusions:**

We first time confirmed that DPYSL2 was a target of HHT and played an oncogene role in AML by regulating JAK/STAT signaling pathway. Therefore, DPYSL2 could serve as a novel prognostic marker and therapeutic target for AML treatment.

## Graphical abstract

DPYSL2 plays an oncogene role in AML and high DPYSL2 expression is an independent prognostic factor for AML patients. Homoharringtonine (HHT) targets DPYSL2 and inhibits the expression of DPYSL2 in AML cells, further reduces the expression of JAK2, STAT3, STAT5, PI3K P85, AKT, GSK3b in JAK/STAT, PI3K/AKT/GSK3b signaling pathways, thus promoting the apoptosis of AML cells.

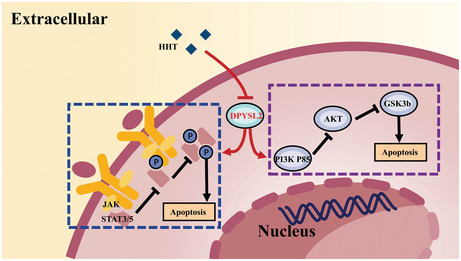

**Abbreviations**
AML: acute myeloid leukemia
BM: bone marrow
CDX: Cell line‐derived xenograft
CR: complete remission
CRMPs: collapsin response mediator proteins
DEGs: differentially expressed genes
DPYSL2: dihydropyrimidinase‐like 2
EFS: event‐free survival
HHT: homoharringtonine
LK: lanthionine ketimine
LSCs: leukemia stem cells
NCG: NOD/ShiLtJGpt‐Prkdcem26Cd52Il2rgem26Cd22/Gpt.
OS: overall survival
PB: peripheral blood
RFS: relapse‐free survival

## 1 INTRODUCTION

Acute myeloid leukemia (AML) is a malignant clonal disorder characterized by blockage of myeloid differentiation and malignant proliferation of immature myeloid blasts in the bone marrow (BM) and peripheral blood (PB).^1^, ^2^ With contemporary therapies, AML is still a disease with a poor prognosis and a high mortality rate: 5 years overall survival (OS) is less than 30%.^3^ Current molecular markers such as prognostic predictors (CEBPα, NPM1, UBA1, FIBA, and PF4 et al.),^4^, ^5^, ^6^ therapeutic predictors (BCL2),^7^, ^8^, ^9^ and predictors with dual prognostic and therapeutic effects (FLT3‐ITD, TP53, IDH1/2)^10^ are critical for precision risk stratification and individualized treatment of AML. Drugs targeting FLT3‐ITD mutations (such as sorafenib)^11^ or BCL2 (such as Venetoclax)^12^ have been widely used in the clinic. However, few studies have thoroughly investigated therapeutic factors, especially those with dual effects.

DPYSL2 (Dihydropyrimidinase‐like 2), also known as DRP2 or CRMP2, is a member of five collapsin response mediator proteins (CRMP) and is involved in axonal growth cone collapse.^13^, ^14^ It encodes a cytosolic protein, which is homologous to the dihydropyrimidinase (DHPase) family in structure.^15^ Previous studies have shown that DPYSL2 promotes neurite outgrowth and modulates signaling processes in the central nervous system.^16^, ^17^ Aberrant expression of DPYSL2 can influence axonal growth^14^ and cause various neurological diseases such as Alzheimer's disease^18^ and schizophrenia.^19^ Recently, two studies illustrated that DPYSL2 exerted an oncologic effect after HOXA11 suppression in glioblastoma^20^ and had an antitumor impact after DPYSL2 inhibition.^21^ However, the function of DPYSL2 in hematopoietic systems or tumors is poorly understood.

Homoharringtonine (HHT) is a cytotoxic alkaloid that was initially extracted from Cephalotaxus hainanensis, and has significant anti‐leukemic effects in AML, chronic myeloid leukemia (CML), and myelodysplastic syndrome (MDS).^22^ In order to investigate the HHT drug resistance mechanisms, we constructed HHT‐resistant AML cell lines via exposure to gradually increasing HHT concentrations. Interestingly, we found that *DPYSL2* was a differentially expressed gene (DEG) between HHT‐resistant and HHT‐sensitive cells by RNA‐seq. In addition, AML patients with high *DPYSL2* expression had poor OS from the results of the public UALCAN database.^23^ Therefore, we speculate that DPYSL2 is a critical regulatory gene in AML pathogenesis and HHT treatment. Identifying its role in AML prognostic significance and biological function is urgently needed.

In the present study, we demonstrated that HHT targeted DPYSL2 in AML cells. Moreover, we identified the expression characteristics of DPYSL2 and explored its function and mechanism in AML pathogenesis and development.

## 2 MATERIAL AND METHODS

### 2.1 Cell lines and patient samples

THP‐1, HL‐60, OCI‐AML3, NB4, Kasumi‐1, KG‐1, and U937 were cultured in RPMI‐1640 medium (Corning Cellgro, USA), MV4‐11, MOLM13, and MV4‐11 luc were cultured in IMDM medium (Corning Cellgro, USA), which were supplemented with 10% Fetal bovine serum (Thermo Fisher Scientific, Gibco, USA) at 37°C with 5% CO_2_. HEK‐293T cells were cultured in DMEM medium supplemented with 10% Fetal bovine serum (Thermo Fisher Scientific, Gibco, USA) at 37°C with 5% CO_2_. HHT resistant cell lines were constructed by increasing the resistance index (RI) in MV4‐11 cells with 10, 30, and 50 nM HHT, and named MV4‐11 R10, MV4‐11 R30, and MV4‐11 R50, respectively.

A total of 198 AML patients with detailed diagnosis and treatment information from March 2010 to June 2016 at the First Affiliated Hospital, College of Medicine, Zhejiang University, were used in this study. Patients with acute promyelocytic leukemia (APL) and those undergoing bone marrow transplantation were excluded. The mononuclear cells of untreated AML patients were isolated by ficoll density gradient centrifugation method and were used in the present study. WHO classification, conventional cytogenetic banding assays, and molecular analyses were performed centrally, as previously described.^24^ We analyzed chromosomal abnormalities and gene mutations of NPM1, FLT3‐ITD, CEBPα, DNMT3A, IDH1, and IDH2 using previously described methods.^25^ The Research Ethics Committee of the First Affiliated Hospital, College of Medicine, Zhejiang University approved this study. All the patients provided written informed consent to participate in the study.

### 2.2 Drugs

HHT (#61847) was purchased from MCE (Med Chem Express, USA).

### 2.3 Quantitative real‐time PCR (qRT‐PCR)

Total RNA was extracted using TRIzol (TAKARA, Japan) and first‐strand complementary DNA synthesis was performed using PrimeScript™ IV 1st strand cDNA Synthesis Mix (TAKARA, Japan). Quantitative PCR was performed in triplicate using the SYBR‐Green PCR Master Mix kit (Takara, Japan) on an IQ5 real‐time PCR instrument (Bio‐Rad, USA). The primers used were as follows:

*DPYSL2* forward:5′‐GTGACTACTCTCTGCATGTGGA‐3′,

reverse: 5′‐TTACCCCGTGATCCTTCACAA‐3′,

*GAPDH* forward: 5′‐GGAGCGAGATCCCTCCAAAAT ‐ 3′,

reverse 5′‐GGCTGTTGTCATACTTCTCATGG ‐ 3′.

### 2.4 Knocking down of DPYSL2 by Short hairpin RNA (shRNA)

To knock down DPYSL2 expression in AML cells, shRNA targeting knockdown (KD) experiments were performed using available sequences targeting DPYSL2, as well as with the non‐targeting control. The shRNA sequences used were as follows.

Control 5′‐ACAGAAGCGATTGTTGATC‐3′,

DPYSL2 sh1 5′‐GGCTTTCAAAGATCGCTTCCA‐3′,

DPYSL2 sh2 5′‐CCTACACATCTATGGGTATCA‐3′.

Briefly, control or targeting shRNA plasmid and package plasmid (psPAX2 and pMG2.G) were co‐transfected into HEK‐293 T cells in a 10 cm cell culture dish with a calcium phosphate cell transfection kit (Beyotime, China). Lentivirus particles were harvested at 48 h and directly added to 1 × 10^6^ cells. The virus‐free medium was changed 24 h after the cells were infected. Infection efficiency was determined by detecting GFP expression using flow cytometry. qRT‐PCR and western blot confirmed decreased mRNA and protein levels of DPYSL2.

### 2.5 Cell viability assay

Cells were seeded in 96‐well plates at 0.5 × 10^5^ for cell growth assay or 1 × 10^5^ for drug sensitivity experiment each well. 20 μl MTS solutions (Promega, Madison, WI) were added to each well, and the cells were incubated for 4 h at 37°C. The plates were read at a wavelength of 490 nm using Varioskan Flash (ThermoFisher, USA).

### 2.6 Flow cytometry analysis

For the apoptosis assay, cells infected with control or DPYSL2 knockdown (DPYSL2‐KD) lentiviral fluid were collected 96 h after infection and stained with PI and Annexin V‐APC for 15 min in 1**×** binding buffer. Then cells were stained with 10 μl PI and 5 μl Annexin‐V for 15 min after 48 h. For cell differentiation analysis, cells were stained with PE‐conjugated CD11b and CD14. For infection efficiency analysis, the cells were infected with GFP labeled virus solution for 72 h, the collected cells were resuspended in 1xPBS, and the expression level of GFP positive cells was detected by flow cytometry FITC channel. The processed cells were analyzed using a NovoCyte D2060R flow cytometer (ACEA, China).

### 2.7 RNA‐sequence and RNA‐seq analysis

The mRNA expression profiles of HHT sensitive strains (MV4‐11 S) and HHT resistant strains with different RIs, including MV4‐11 R10/R30/R50, were obtained using high‐throughput sequencing (RNA‐Seq). The sequenced data were obtained using the Illumina HiSeq X Ten platform. Then data have been uploaded to NCBI's SRA and access to cite for these SRA data: PRJNA664675. For RNA‐Seq data, mRNA expression levels were calculated as reads per kilobase per million reads. The DE Seq (1.18.0) R package was used to analyze differentially expressed mRNAs. For DPYSL2 KD RNA‐seq, RNA was isolated from MV4‐11 cells 72 hours after transduction with shRNA targeting DPYSL2 or control and was sequenced (Illumina HiSeq X Ten platforms). The data have been uploaded to NCBI's SRA and access to cite for these SRA data: PRJNA699422. KEGG analysis was used to explore the regulatory pathways before and after the DPYSL2 KD.

### 2.8 GSEA analysis

The original AML data were downloaded from the TCGA website. The patients' gene expression profiles and clinical information were extracted and integrated using the R language. The enrichment of the gene set between DPYSL2 low and high groups was analyzed using GSEA software (version 3.0).

### 2.9 Auto‐docking

Auto‐Dock software (version 4.0) and PyMOL were used for docking between DPYSL2 and HHT. The structural file of DPYSL2 was downloaded from the PDB. The DPYSL2 structure, numbered 5LXX (http://www1.rcsb.org/structure/5LXX) in the PDB database, was used in this study. The structure of HHT was downloaded from PubChem (https://pubchem.ncbi.nlm.nih.gov/). The confirmed DPYSL2 small molecule ligand BTB^26^ served as a positive control to evaluate the binding capacity and binding site of HHT with DPYSL2. The bis–tris pocket was used for docking to reveal the binding activity between HHT and DPYSL2 in our study.

### 2.10 Western blot

Cells were harvested, and 1 **×** RIPA buffer (Thermo Fisher Scientific, USA) was added to the cells on ice for 15 min. Cells were lysed with ultrasound and centrifuged at 12,000 g for 15 min at 4°C to pellet cell debris. The protein concentration was determined using a BCA reagent (Thermo Fisher Scientific, USA). After heating to 100°C for 10 min, the protein samples were separated by SDS‐PAGE gel and transferred to PVDF membranes (Millipore, Burlington, MA). The membrane was blocked with 5% powder milk and probed with an antibody at 1:1000 overnight. After washing with TBST buffer, the membranes were incubated with horseradish peroxidase‐conjugated secondary antibodies (1:5000). The target proteins were visualized using an ECL kit (Thermo Fisher Scientific, USA) and imaged using a Bio‐Rad imaging system. The antibodies were summed in Table S1.

### 2.11 Cell line‐derived xenograft (CDX) Mice model

NCG (NOD/ShiLtJGpt‐Prkdcem26Cd52Il2rgem26Cd22/Gpt) mice were raised at the Experimental Animal Center of Zhejiang Chinese Medicine University Laboratory Animal Research Center and were approved by the Ethics Committee for Laboratory Animals. Animal care was performed following the institutional guidelines. MV4‐11‐luc cells were infected with DPYSL2 control or DPYSL2 KD lentiviral solution, and 1 × 10^6^ cells were injected into six‐week‐old NCG mice via the tail vein. Cell engraftment was assessed by intraperitoneal injection of luciferase (100 mg/kg), followed by imaging using an IVIS Lumina LT system (PerkinElmer, CA, USA). Leukemic burden and body weight of mice were assessed by bioluminescence imaging every 7 days. The mice were euthanized when their lower limbs were paralyzed. The peripheral blood and bone marrow of mice were used to measure the expression of humanized CD45. The survival data were summed when the mice were sacrificed.

### 2.12 Statistics

The primary endpoints of the study were OS, EFS, and RFS. OS was defined as the time from diagnosis until death due to any cause or last follow‐up. EFS was defined as the time from diagnosis until its removal from the study due to incomplete remission, relapse, or death. RFS was defined as the time from the date of complete remission (CR) to relapse or death. The OS, EFS, and RES survival rates were analyzed using Kaplan–Meier curve analysis with IBM SPSS Statistics 20 software. Multivariate analysis of the prognostic significance of DPYSL2 was performed using the Cox regression method with the IBM SPSS Statistics 20 software.

For experimental analysis, except for mice experiment in vivo and RNA sequence experiment, other experiments were conducted for three times independently. unpaired two‐tailed Student's *t*‐test or analysis of variance (ANOVA) and Student's *t*‐test were performed using the IBM SPSS Statistics 20 software. NS not significant, *p <* 0.05*, *p <* 0.01**, *p* < 0.001***.

## 3 RESULTS

### 3.1 HHT targets DPYSL2 in AML treatment

**FIGURE 1:**
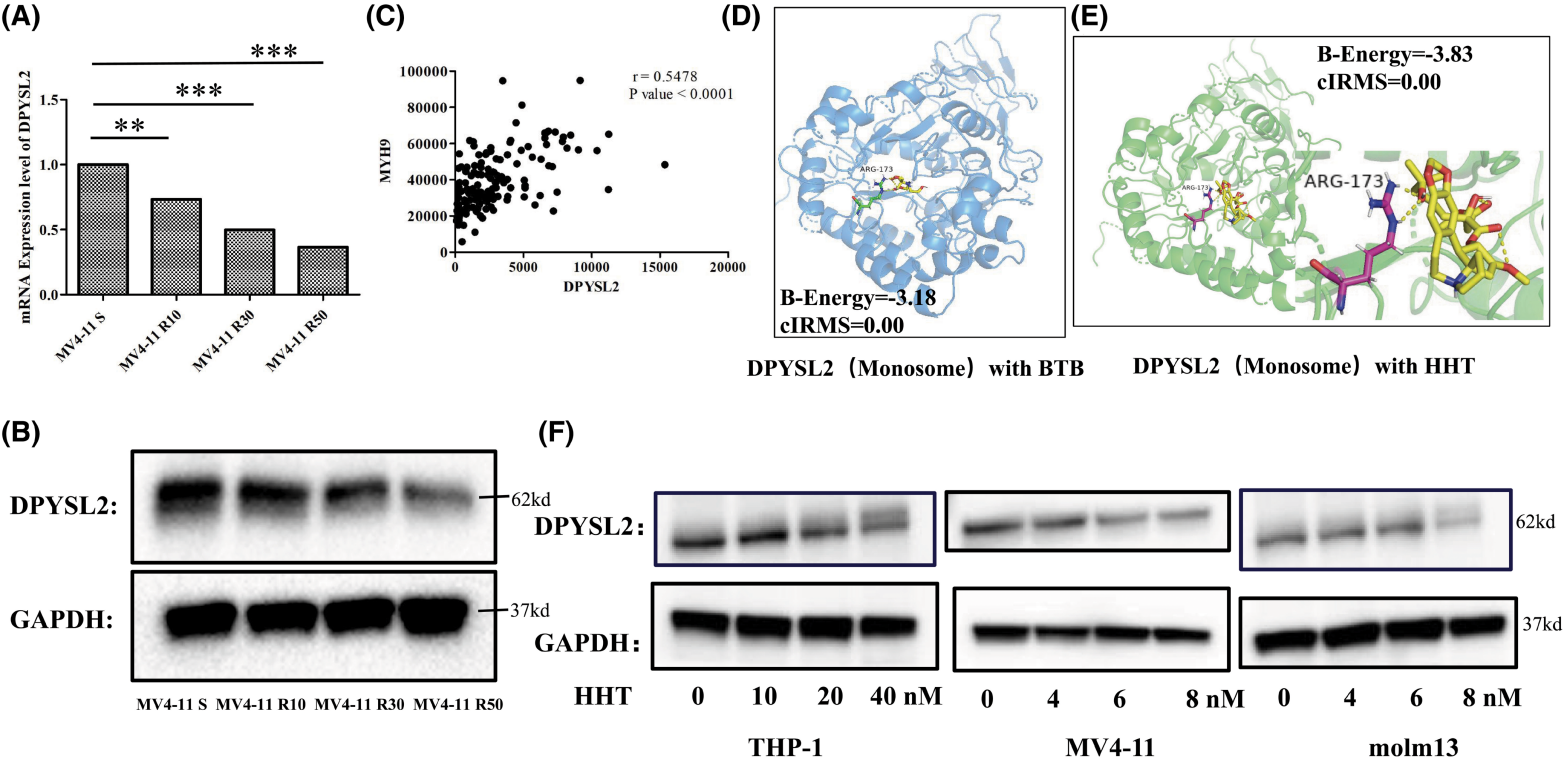
HHT targets DPYSL2 in AML treatment. (A) DPYSL2 expression levels among MV4‐11 S, MV4‐11 R10, MV4‐11 R30, MV4‐11R50 were identified by RNA‐seq. (B) The protein expression level among MV4‐11 S, MV4‐11 R10, MV4‐11 R30, MV4‐11R50 were detected by western‐blot. (C) The correlation of the mRNA expression level between DPYSL2 and HHT direct target MYH9. The expression data were from TCGA cohort. (D) The binding efficacy of BTB in the bis‐tris site of DPYSL2 by auto‐dock, which worked as a positive control. (ARG‐173 was represent as green, BTB was represent as yellow). (E) The binding efficacy of HHT in the bis‐tris site of DPYSL2 by auto‐dock (ARG‐173 was represent as red, HHT was represent as yellow). (F) The expression level of DPYSL2 in AML cells treated by different concentrations of HHT after 48 h was measured by western‐blot. *p <* 0.01**, *p* < 0.001***.

To explore the differential of mRNA expression profiles between HHT sensitive strains and HHT resistant strains, the RNA sequences of MV4‐11 sensitive cells were compared with MV4‐11 R10, MV4‐11 R30 and MV4‐11 R50 cells separately. The results showed that the transcriptomes between HHT resistant and sensitive cells were considerably divergent (Figure S1A, B). Of these, DPYSL2 was significantly decreased in HHT resistant cells compared to sensitive cells, both at the mRNA and protein levels (Figure 1A, B). GO enrichment analysis revealed that DPYSL2 was one of the crucial genes in three of the top 30 pathways (GO:0005515, GO:0005874, GO:0042802) both between sensitive cells and MV4‐11R30, and between sensitive cells and MV4‐11R50 cells. Furthermore, we found DPYSL2 was significantly positively correlated with MYH9, a confirmed HHT target gene by our group^27^ (Figure 1C), indicating that DPYSL2 may be a target of HHT.

Then auto‐docking was performed to explore the relationship between DPYSL2 and HHT. The high‐resolution crystal structure and small‐molecule binding pocket of DPYSL2 were confirmed by Matti Myllykoski.^26^ As shown in Figure 1D, the ARG‐173 residue in the pocket site can form a hydrogen bond with BTB, a confirmed small‐molecule ligand of DPYSL2, with a binding energy of −3.18, and a binding coefficient of 0.00. HHT could also form a hydrogen bond with the ARG‐173 residue with lower binding energy (−3.83) (Figure 1E), indicating stronger binding activity.

Next, we explored whether HHT could inhibit DPYSL2 expression. After HHT treatment, DPYSL2 protein levels decreased in the three AML cell lines (Figure 1F). Our research is the first to reveal that HHT can target DPYSL2 in leukemia treatment.

### 3.2 High expression of DPYSL2 was an independent poor prognostic factor of AML

**FIGURE 2:**
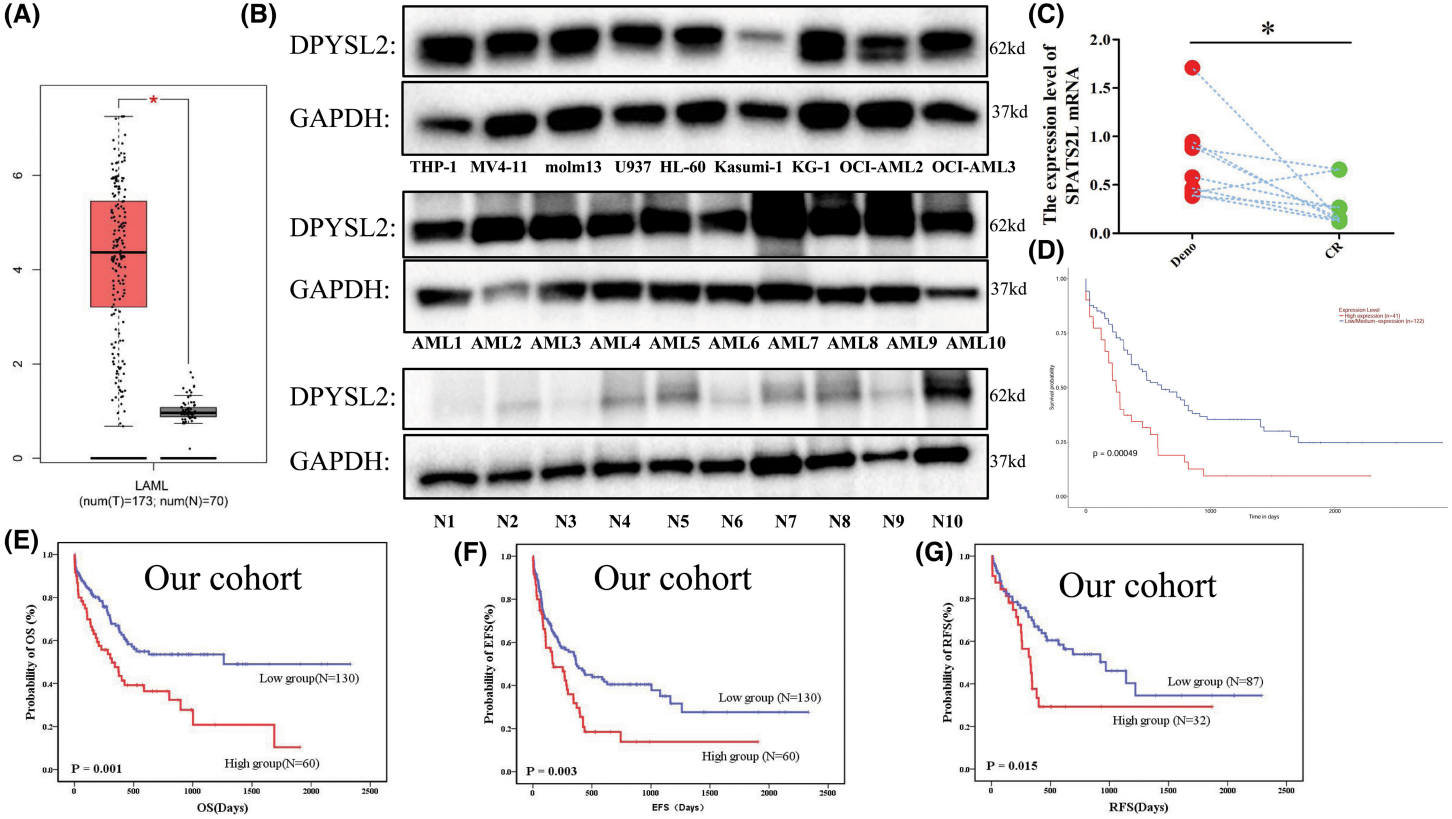
High expression of DPYSL2 was an independent poor prognostic factor of AML. (A) The mRNA expression level of DPYSL2 in 173 AML samples (red) and 70 normal samples (gray) from GEPIA(http://gepia.cancer‐pku.cn/). (B) The protein expression level of DPYSL2 from nine AML cell lines, ten primary AML patients(AML1 ~ AML10), and ten health donors(N1 ~ N10) by western‐blot. (C) The expression of DPYSL2 in AML patients before treatment and after remission by RT‐qPCR. (D) The OS of AML patients from TCGA cohort between DPYSL2‐low group (blue) and DPYSL2‐high group (red). (E) The OS of AML patients from our cohort between DPYSL2‐low group (blue) and DPYSL2‐high group (red). (F) The EFS of AML patients from our cohort between DPYSL2‐low group (blue) and DPYSL2‐high group (red). (G) The RFS of AML patients from our cohort between DPYSL2‐low group (blue) and DPYSL2‐high group (red). *p* < 0.05*.

As DPYSL2 is one of the targets of HHT in leukemia treatment, we then explore the role of DPYSL2 in AML pathogenesis. We found AML patients had higher DPYSL2 mRNA levels than normal samples in the public database GEPIA (Figure 2A), and had higher protein levels in AML cell lines and AML patients than healthy donors (Figure 2B). Of note, dynamic mRNA expression level demonstrated that the mRNA level of DPYSL2 was reduced after CR in DPYSL2 high‐diagnosis AML patients (Figure 2C). These results indicated that DPYSL2 was overexpressed in AML cell lines and AML patients and may have prognostic significance and treatment response for AML.

To further analyze whether DPYSL2 could be a prognostic marker for AML, patients with AML from two independent cohorts were used for survival analysis. A total of 163 AML patients from TCGA database showed that AML‐M5 (acute monocytic leukemia) patients had higher DPYSL2 expression than other types of AML (Figure S2A‐B) and patients with high DPYSL2 expression had shorter OS (*p* = 0.00049) (Figure 2D). Patients from our center were used to validate the public database results. The clinical characteristics of the 190 patients with high and low DPYSL2 expression levels are presented in Table 1. Consistent with the results of the TCGA database, higher mRNA DPYSL2 expression levels were also found in AML‐M5 patients in our cohort (Figure S2C‐D), and patients with high DPYSL2 expression had a shorter OS (*p* = 0.001) (Figure 2E) and a worse EFS (*p* = 0.003) (Figure 2F) and RFS (*p* = 0.015) (Figure 2G). Further multivariate analysis demonstrated that high DPYSL2 expression in AML was an independent marker of poor prognosis for OS (*p* = 0.027), EFS (*p* = 0.040), and RFS (*p* = 0.050) (Table 2). These results indicated that high DPYSL2 expression is an independent poor prognostic factor for AML.

**TABLE 1:** Characteristics of AML patients by high and low DPYSL2 expression

Variables	Low group	High group	*p* value
Number, *n* (%)	131(69%)	61(31%)	
Age, median (range)	47(15,78)	49(17,82)	0.312
Female, *n* (%)	60(43.8)	20(32.8)	0.160
WBC, median (range)	35.10(0.8336.9)	41.06(0.2354)	0.178
HB, median (range)	82.1(35,153)	88.4(37.4134)	0.661
PLT, median (range)	40(4778)	46(2776)	0.204
BM blast, median (range)%	72(15,96.65)	74(23,94.5)	0.369
FAB type			0.001
M0	9(6.6)	4(6.6)	
M1	17(12.4)	7(11.5)	
M2	68(49.6)	13(21.3)	
M4	8(5.8)	5(8.2)	
M5	27(19.7)	29(47.5)	
M6	6(2.9)	1(1.6)	
Karyotype risk, *n* (%)			0.359
Favorable	7(5.1)	1(1.6)	
Intermediate	117(85.4)	52(85.2)	
Unfavorable	11(8.0)	2(3.3)	
Gene mutation			
FLT3‐ITD	28(20.4)	9(14.8)	0.327
NPM1	37(27.0)	13(21.3)	0.376
CEBPA^DM1^	18(13.1)	5(8.2)	0.339
IDH1	9(6.6)	4(6.6)	1.000
IDH2	8(5.8)	2(3.3)	0.506
DNMT3A	9(6.6)	9(14.8)	0.117
Treatment protocols^a^			0.123
DA	78(56.9)	35(57.4)	
IA	32(23.4)	16(26.2)	
HAA	18(13.1)	2(3.3)	

Abbreviation: DM, Double‐allele.

^a^ The protocols used for induction therapy in different groups including daunorubicin/Ara‐C (DA)‐based treatment group, idarubicin/Ara‐C (IA)‐based, and homoharringtonine/Ara‐C/aclarubicin (HAA)‐based treatment group.

**TABLE 2:** Multivariate analysis of the prognostic significance of DPYSL2 on AML from our cohort^a^

	*p* value	HR (95%CI)
OS	0.027	1.781(1.006 ~ 2.974)
EFS	0.040	1.611(1.021 ~ 2.542)
RFS	0.050	1.968(1.001 ~ 3.869)

Abbreviations: CI, confidence interval; HR hazard ratio.

^a^ Multivariate analysis were performed by Cox proportional hazards models with the backward likehood stepwise procedures.

### 3.3 Inhibition of DPYSL2 could suppress cell growth and induce apoptosis of AML cells

**FIGURE 3:**
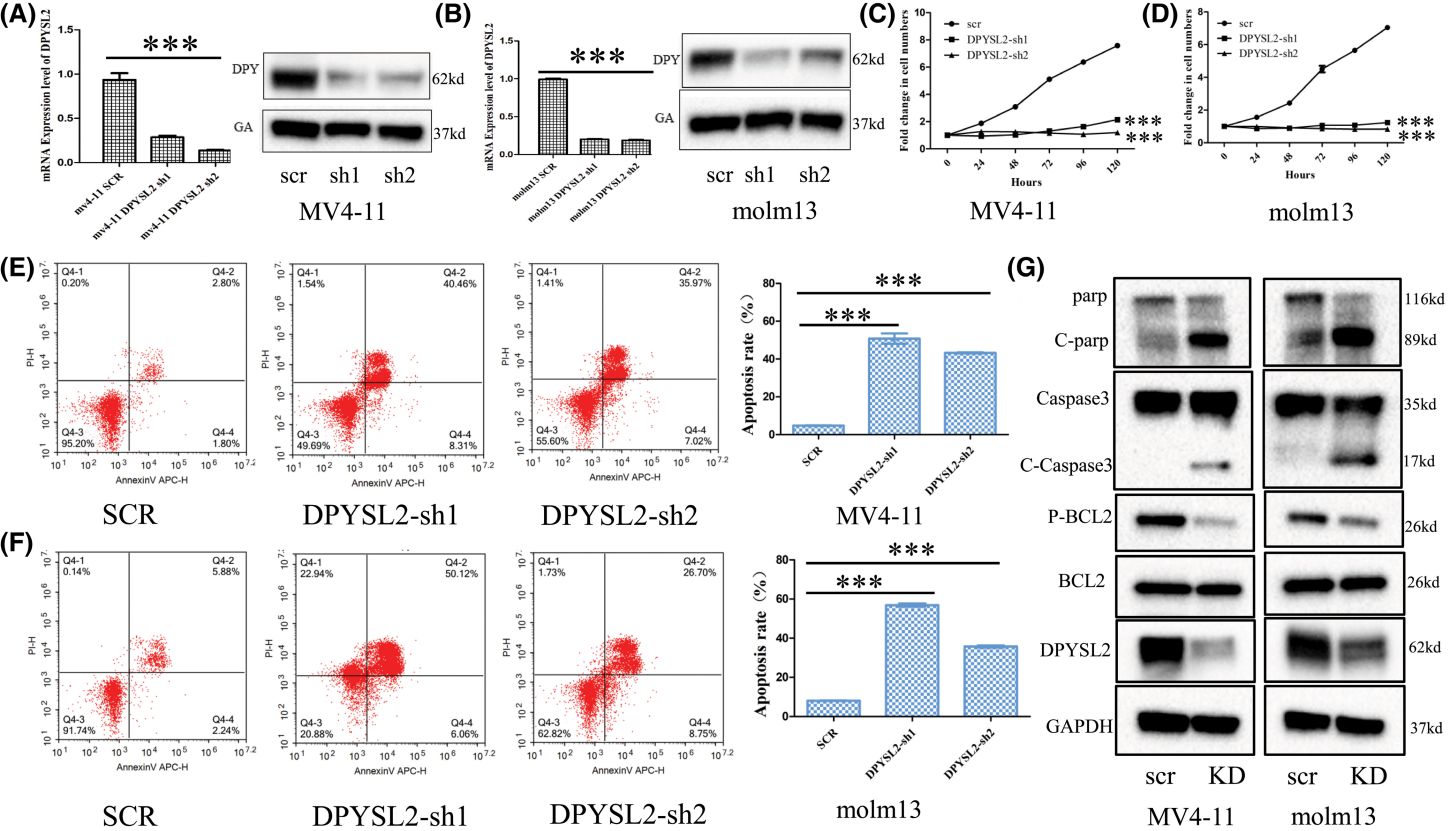
The regulation function of DPYSL2 in AML cells in vitro. (A‐B) The decreased level of DPYSL2 in AML cells by shRNA targeting KD was confirmed by RT‐qPCR and western‐blot. (C‐D) The growth curve of AML cells with or without DPYSL2 KD was measured by MTS. (E‐F) The apoptosis of AML cells with or without DPYSL2 KD was measured by Flow‐cytometry. (G) The apoptosis‐related proteins were measured by western‐blot. NS not significance, *p* < 0.001***.

Next, we sought to identify the regulatory role of DPYSL2 in the growth and viability of AML cells via shRNA‐targeted knockdown. The GFP fluorescence rate inferred that AML cells transfected with lentivirus exceeded 90% (Figure S3A, B). RT‐qPCR and western blot results confirmed the inhibitory effect of DPYSL2 by transfection with sh‐DPYSL2 (Figure 3A, B). We found that knockdown of DPYSL2 suppressed the growth of three AML cell lines (MV4‐11, MOLM13, and THP‐1 cells) (Figure 3C, D and Figure S3C,D) and induced apoptosis in MV4‐11 and MOLM13 cells (Figure 3E, F). However, there was no effect on the differentiation of AML cells (Figure S3E, F). In addition, knockdown of DPYSL2 promoted the cleavage of the apoptosis regulatory proteins PARP and caspase3, and inhibited the expression of phosphor‐BCL2 without affecting total BCL2 expression (Figure 3G). These findings indicated that DPYSL2 plays an oncogenetic role in AML pathogenesis and may be a potential candidate target for AML drug treatment.

### 3.4 Inhibition of DPYSL2 could prolong the survival of AML CDX mice

**FIGURE 4:**
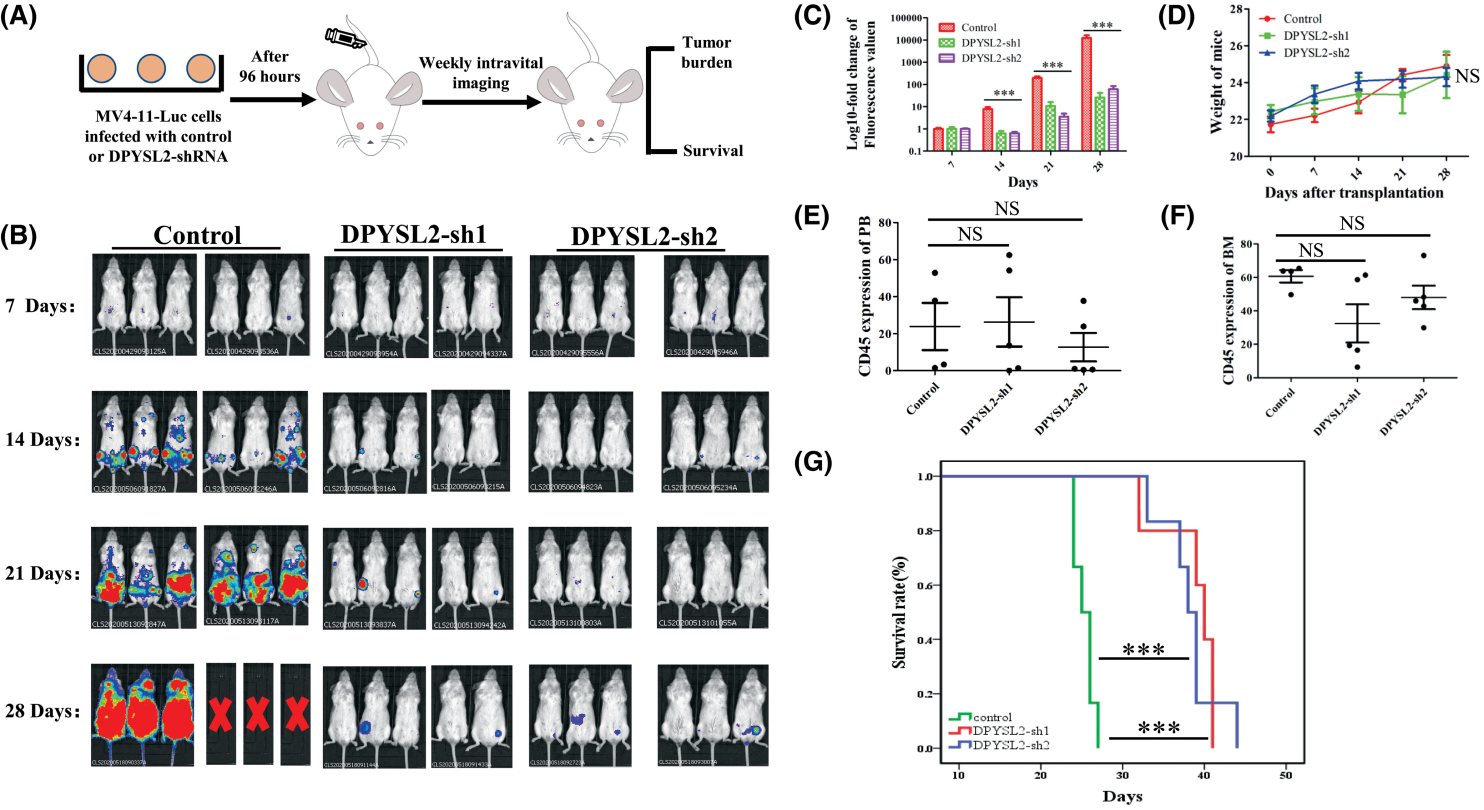
The regular function of DPYSL2 in AML cells in vivo. (A) Schematic illustration of the MV4‐11 luc AML xenograft NCG model coupled with control and shRNA targeting DPYSL2 KD. (B) In vivo imaging results of the abdomen of mice on the 7th, 14th, 21st, and 28th day after cell injection. (C) Quantitative analysis results of in vivo imaging results of the abdomen of mice. (D) The body weight of mice among the three groups. (E‐F) The engraftment ratio of leukemic cells into PB (E) and BM(F) at the endpoint of the three groups was measured by detecting the human CD45. (G) Kaplan–Meier curves of the three groups xenotransplanted with human MV4‐11 luc AML cells. NS not significance, *p* < 0.001***.

To further confirm the function of DPYSL2 in AML, we conducted in vivo experiments using an AML CDX model. The experimental design is illustrated in Figure 4A. Abdominal imaging showed that mice in the DPYSL2 control group had a higher tumor burden than those in the DPYSL2‐sh1 and DPYSL2‐sh2 groups (Figure 4B, C). Moreover, the dorsal view results were consistent with the abdominal imaging results (Figure S4A‐B). In the terminal stage of mice, there was no significant difference in body weight and humanized CD45 expression in PB and BM among the groups (Figure 4D, F). However, mice in the DPYSL2 sh1 and DPYSL2 sh2 groups had more prolonged survival than the control group (*p* < 0.001) (Figure 4G). These animal experiments suggested that inhibition of DPYSL2 could prolong the survival of AML NCG mice.

### 3.5 DPYSL2 targeted JAK2/STAT3/5‐PI3K P85/AKT/GSK3b axis in AML

**FIGURE 5:**
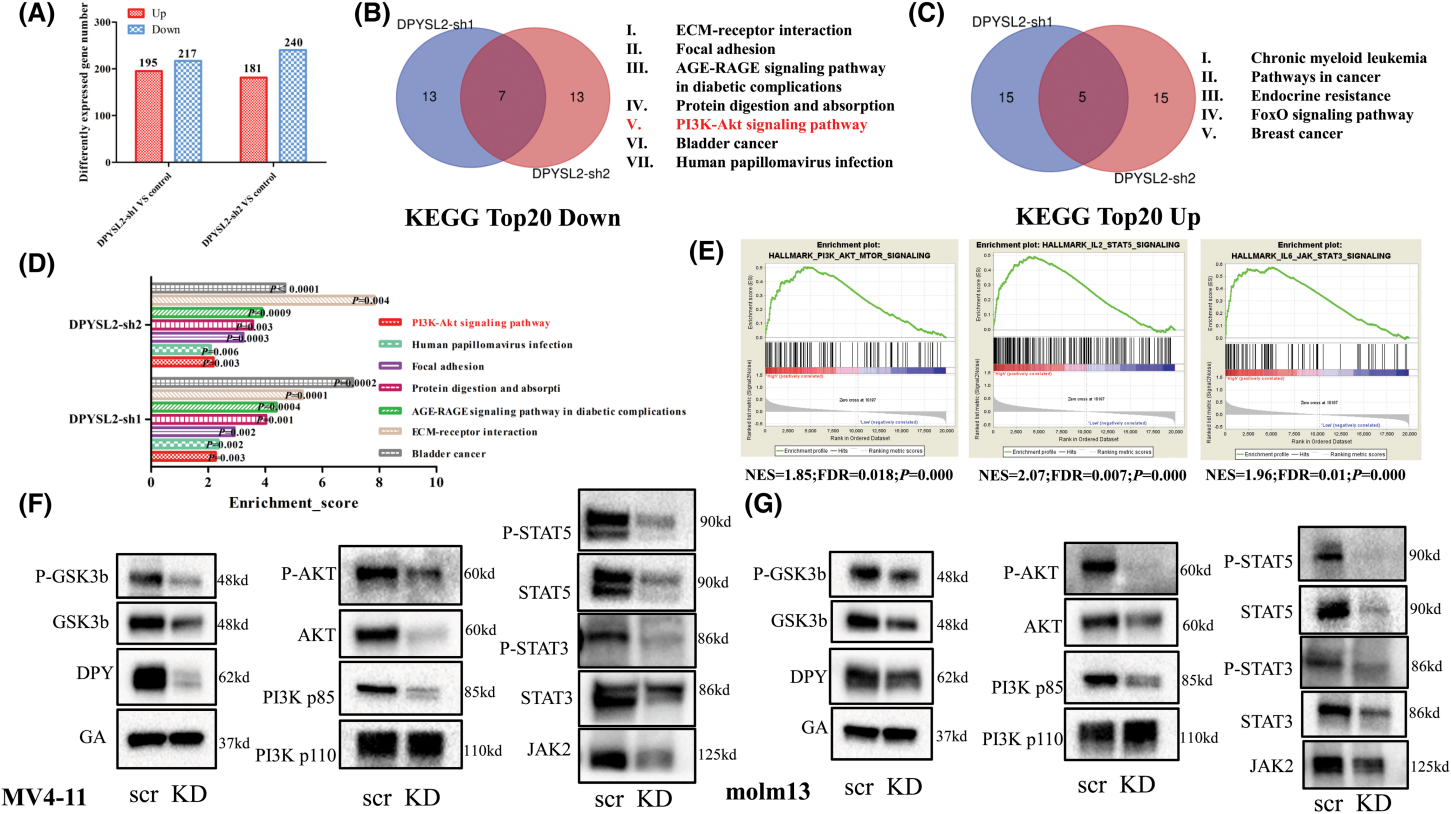
The regulation mechanism of DPYSL2 in AML. (A) The significant DEGs of DPYSL2 sh1 or DPYSL2 sh2 compared with control by RNA‐seq, respectively. (B) KEGG enrichment analyzed the top 20 pathways of DPYSL2 sh1 or DPYSL2 sh2. The Venn diagram showed the pathways that were jointly inhibited after DPYSL2 KD by sh1 or sh2. (C) The Venn diagram showed the pathways that were jointly activated after DPYSL2 KD by sh1 or sh2. (D) Normalized enrichment score (NES) and p value of the seven pathways. D The PI3K‐AKT–MTOR pathway, IL6‐JAK‐STAT3 pathway, IL2‐STAT5 pathway were activated in cells from DPYSL2 high group patients by GSEA. (E‐F) The change of proteins, including JAK/STAT3/STAT5/PI3K/AKT/GSK3b, were measured by western‐blot.

To explore the molecular mechanism of DPYSL2 in AML, we examined global changes in gene expression using RNA sequencing in MV4‐11 cells transfected with DPYSL2 control or shRNA. The gene expression profile was considerably divergent between the control and DPYSL2 KD cells (Figure S5A‐D). All DEGs were subjected to KEGG enrichment analysis (Figure 5A). As for the top 20 pathways, seven pathways from downregulated DEGs were enriched in both DPYSL2 sh1 and DPYSL2 sh2 compared with the control (Figure 5B). In comparison, five pathways from upregulated DEGs were enriched (Figure 5C). The PI3K‐AKT pathway, which belonged to the top 20 downregulated pathways, was suppressed in both DPYSL2 sh1 and DPYSL2 sh2 cells (Figure 5D). In addition, gene expression profiles were also significantly different between DPYSL2‐high and DPYSL2‐low patients from TCGA database (Figure S5E). The top 20 pathways are presented in Table S2. The PI3K‐AKT pathway was also enriched (Figure 5E). Interestingly, the top five pathways were related to immune regulation, two of which belonged to the JAK/STAT, IL2‐STAT5, and IL6‐STAT3 pathways (Figure 5E). Therefore, we assumed that the JAK2/STAT3/STAT5/p‐STAT5/PI3K P85/AKT/p‐AKT/GSK3b axis is regulated by DPYSL2 in AML.

We then compared the protein levels of this axis between the control and KD AML cells. As expected, the essential proteins JAK2/STAT3/STAT5/p‐STAT5/PI3K P85/AKT/p‐AKT/GSK3b decreased after DPYSL2‐KD in AML cells, while the expression of PI3K P110 did not change (Figure 5F). Therefore, we speculate that DPYSL2 plays an oncogene role in AML by regulating the JAK2/STAT axis.

## 4 DISCUSSION

**FIGURE 6:**
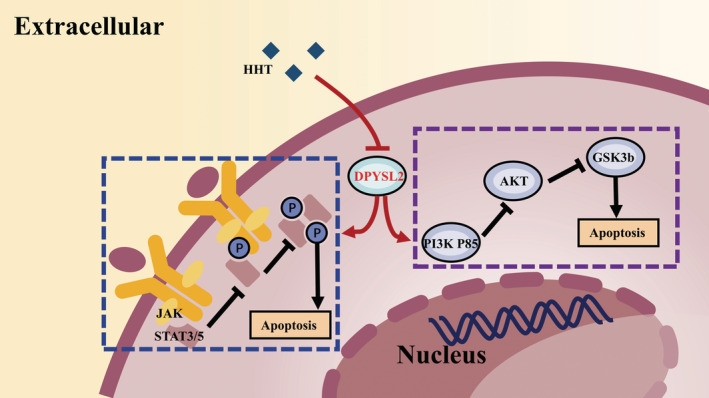
The schematic diagram of DPYSL2's regulation of AML. Homoharringtonine (HHT) targets DPYSL2 and inhibits the expression of DPYSL2 in AML cells, further reduces the expression of JAK2, STAT3, STAT5, PI3K P85, AKT, GSK3b in JAK/STAT, PI3K/AKT/GSK3b signaling pathways, thus promoting the apoptosis of AML cells.

The present study first time confirmed that DPYSL2 plays an oncogene role in AML and could be a novel therapeutic target and prognostic marker. First, DPYSL2 was decreased in HHT resistant cell lines, and was a target of HHT. Second, DPYSL2 expressed at higher levels in AML cell lines and patients than in healthy donors, and a high expression level of DPYSL2 was an independent poor prognostic factor for OS, EFS, and RFS in AML patients. Third, DPYSL2 plays an oncogene role in the development of AML. Leukemic growth of AML cells was dependent on high DPYSL2 expression, both in vitro and in vivo. Fourth, the inhibition of DPYSL2 suppressed the JAK2/STAT3/STAT5‐PI3K P85/AKT/GSK3b axis and further induced apoptosis in AML cells. A schematic diagram of DPYSL2 regulation in AML is shown in Figure 6.

HHT based “HAA” program (HHT plus aclarubicin and cytarabine) is the first‐line therapy for non‐elder AML patients in China.^28^, ^29^ Our group has demonstrated that myosin‐9 was the target protein of HHT and played an important role in the HHT‐induced apoptosis of leukemia cells.^27^ Another study showed NKRF, an NF‐κB–repressing factor, was targeted by HHT.^30^ With the widespread use of HHT, identification of direct binding protein of HHT is important for individualized therapy. The present study confirmed HHT could directly target DPYSL2 by auto‐docking, which is an effective way to reveal the interaction between small molecules and proteins.^31^

DPYSL2 has emerged as a potential drug target for treating neurological diseases.^15^ Lacosamide,^32^ lanthionine ketimine (LK), and its 5‐ethyl ester (LKE)^33^ were confirmed drugs that directly target DPYSL2 to treat partial‐onset seizures in adults. However, up to now, the role and mechanism of DPYSL2 in AML is little konwon. Only Noura et al. showed that exogenous expression of DPYSL2A resulted in the monocytic differentiation of THP‐1 cells and suppressed cell growth.^34^ So they thought that DPYSL2A might serve as a tumor suppressor gene. Surprisingly, our research results are inconsistent with the existence of Noura et al. We found that DPYSL2 changes did not affect the differentiation of THP‐1 cells and had oncogene effects in AML. This may be because Adachi et al. focused on the peer isomer DPYSL2A of DPYSL2. It also suggests that DPYSL2 may play a dual role in AML, which needs further study.

A previous study showed that the PI3k/Akt/GSK‐3b/CRMP‐2 (DPYSL2) pathway plays a critical role in neuronal differentiation.^16^, ^35^ In addition, the 5′‐UTR of DPYSL2 interacts with mTOR effectors in schizophrenia.^19^ As mTOR is downstream of the PI3K/AKT pathway,^36^ we inferred that DPYSL2 is a crucial regulator of the PI3K/AKT pathway. Constitutive activation of the PI3K/AKT/mTOR pathway has been detected in 50%–80% of AML patients and is associated with poor OS.^37^, ^38^ PI3K/AKT/mTOR was confirmed to be a critical oncogenic signaling pathway in AML leukemia stem cells (LSCs). Persistent LSC populations underlie patient relapse and the development of therapy resistance.^39^, ^40^ Targeting the critical components of this pathway may represent an effective treatment for AML LSCs. In the present study, we found that the PI3K/AKT pathway was suppressed after DPYSL2 knockdown. Furthermore, DPYSL2 single‐gene GSEA analysis of AML data from TCGA database revealed a strong activation of the PI3K/AKT/ mTOR pathway in patients in the DPYSL2 high expression group. Two of the top five pathways enriched by GSEA were IL2‐STAT5 and IL6‐STAT3, which are upstream of the PI3K/AKT/mTOR pathway. These results indicate that DPYSL2 may play a regulatory role in AML through the JAK/STAT‐PI3K/AKT axis. In the present study, the expression of critical proteins in JAK2/STAT3/STAT5‐PI3K P85/AKT/GSK‐3b decreased after DPYSL2 knockdown in AML cells. DPYSL2 also regulates the upstream and downstream pathways of the PI3K/AKT pathway in AML.

In summary, our results suggest that DPYSL2 plays an oncogene role in AML and is the target of HHT for treating AML. Therefore, DPYSL2 may be a promising marker for evaluating AML and an effective target for treating AML, which is of great significance to improving the AML prognosis system and the targeting of DPYSL2 to treat AML in the future.

## AUTHOR CONTRIBUTIONS

**Fenglin Li:** Formal analysis (lead); methodology (equal); software (lead); writing – original draft (lead). **Qing Ling:** Conceptualization (equal); investigation (lead); software (equal); validation (equal). **Jiaying Lian:** Data curation (equal); resources (equal); validation (equal). **Ying Chen:** Conceptualization (equal); investigation (equal); methodology (equal); writing – original draft (supporting). **Chao Hu:** Data curation (equal); resources (equal); visualization (supporting); writing – review and editing (equal). **Min Yang:** Conceptualization (equal); data curation (equal); resources (equal); writing – review and editing (supporting). **Xiang Zhang:** Formal analysis (supporting); supervision (equal); visualization (supporting); writing – review and editing (supporting). **Chenying Li:** Formal analysis (supporting); investigation (supporting); writing – review and editing (equal). **Shihui Mao:** Formal analysis (supporting); methodology (equal); software (supporting). **Wenle Ye:** Supervision (equal); validation (equal); visualization (equal). **Xia Li:** Data curation (supporting); methodology (supporting); validation (supporting); writing – original draft (supporting). **Xiangjie Lin:** Resources (supporting); software (equal); validation (equal). **Wenwen Wei:** Conceptualization (equal); formal analysis (supporting); investigation (supporting); writing – review and editing (supporting). **Xin Huang:** Conceptualization (equal); methodology (supporting); resources (supporting). **Jiajia Pan:** Data curation (supporting); formal analysis (supporting); software (supporting). **Yu Qian:** Methodology (supporting); resources (equal); writing – original draft (supporting). **Jinghan Wang:** Resources (supporting); software (supporting); visualization (supporting). **Ying Lu:** Funding acquisition (equal); investigation (equal); project administration (lead); writing – review and editing (equal). **Jie Jin:** Conceptualization (lead); funding acquisition (lead); investigation (lead); project administration (equal); writing – review and editing (lead).

## FUNDING INFORMATION

This work was supported by grants from the National Natural Science Foundation of China (81820108004) and the Ningbo Science and Technology Project (2019C50071).

## CONFLICT OF INTEREST

The authors have no conflict of interest.

## ETHICS APPROVAL AND CONSENT TO PARTICIPATE

The Research Ethics Committee of the First Affiliated Hospital, College of Medicine, Zhejiang University approved this study.

## Supporting information


Data S1.

## Data Availability

Data sharing is not applicable to this article as no new data were created or analyzed in this study.
