# Supplementary material for: Dihydropyrimidinase‐like 2 can serve as a novel therapeutic target and prognostic biomarker in acute myeloid leukemia
Source: Cancer Med. 2023 Jan 9;12(7):8319–30. doi: 10.1002/cam4.5531 (PMC10134331; doi:10.1002/cam4.5531)
Supplement: Supplementary file 1 — Data S1. [file CAM4-12-8319-s001.pdf]

Supplementary materials:

Supplementary Figures and legends:

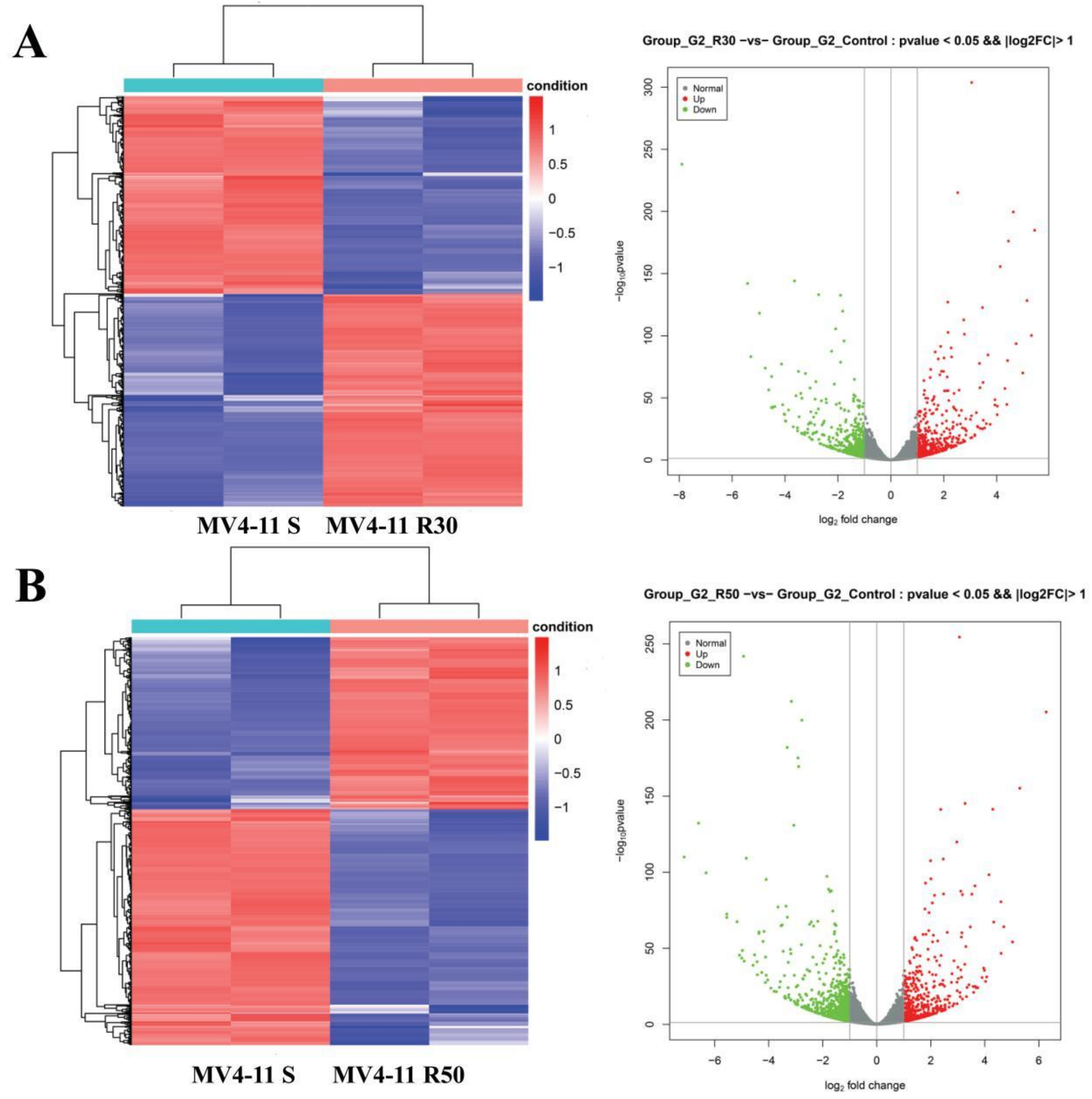

FigureS1 DPYSL2 significant decreased in HHT resistant cells compared with HHT sensitive cells. (A) The different expression genes(DEGs) between MV4-11S and MV4-11 R30 cells displayed by heat map and Volcano through analyzing the RNA-Seq data. (B) The different expression genes(DEGs) between MV4-11S and MV4-11 R50 cells displayed by heat map and Volcano through analyzing the RNA-Seq data.

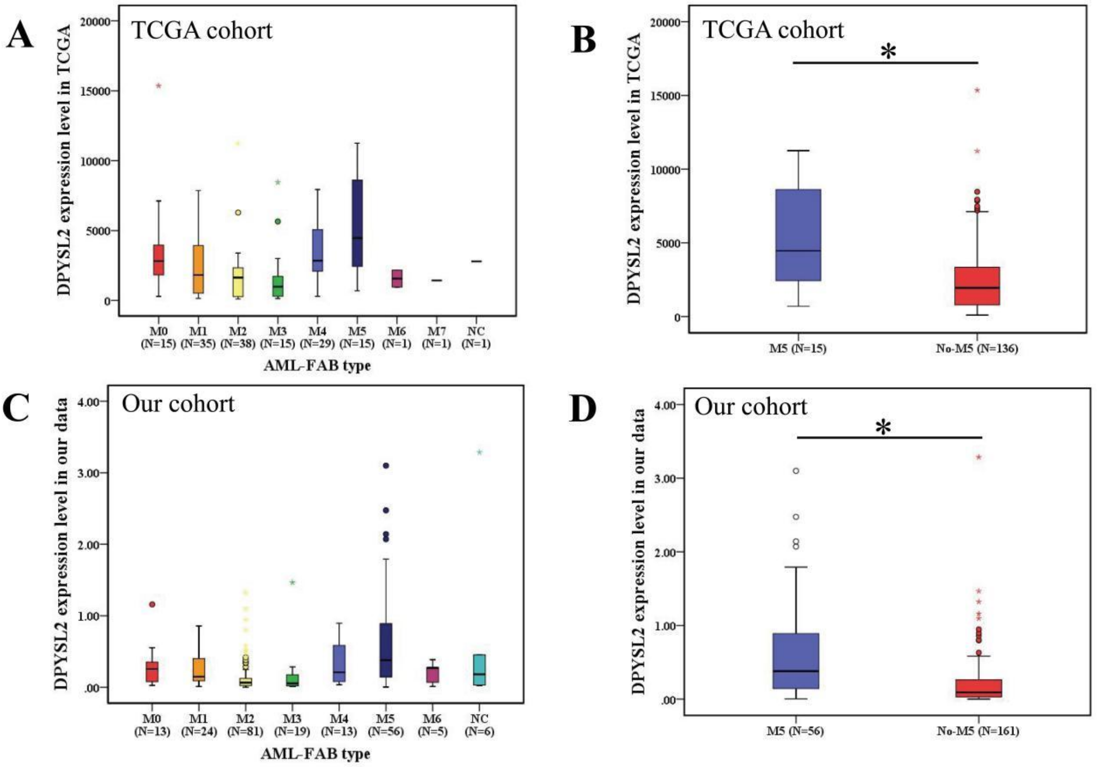

FigureS2 The expression characteristics of DPYSL2 in AML FAB type (A) The expression level of DPYSL2 in AML FAB type from TCGA database. (B) The expression level of DPYSL2 between AML-M5 and non AML-M5(No-M5) from TCGA database. (C) The expression level of DPYSL2 in AML FAB type from our cohort. (D) The expression level of DPYSL2 between AML-M5 and non AML-M5 (No-M5) from our cohort. NS not significance $P < 0.05$ *

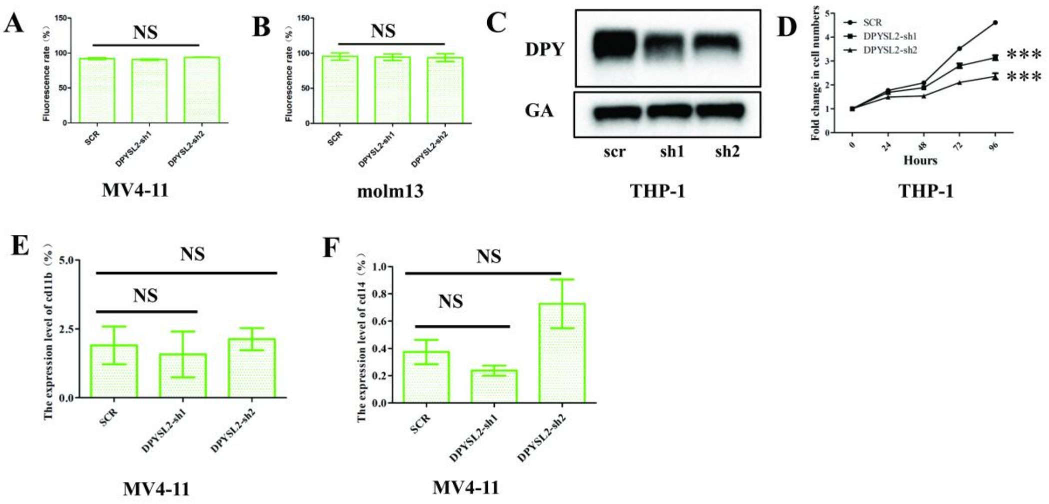

FigureS3 The transfection rate and The growth and differentiation after DPYSL2 KD in AML cells. (A-B) MV4-11 and molm13 were transfected with lentivirus for 72 hours to determine the transfection efficiency by detecting the GFP expression rate by flow cytometry. (C) The decreased level of DPYSL2 in THP-1 cells after being transfected with lentivirus for 96 hours by western-blot. (D) The growth curves of THP-1 cells with or without DPYSL2 KD were measured by MTS solution starting 96 hours after transfection. (E-F) The differentiation antigen CD11b, CD14 of MV4-11 cells after transfection were detected by flow cytometry. NS not significance , P < 0.001 ***

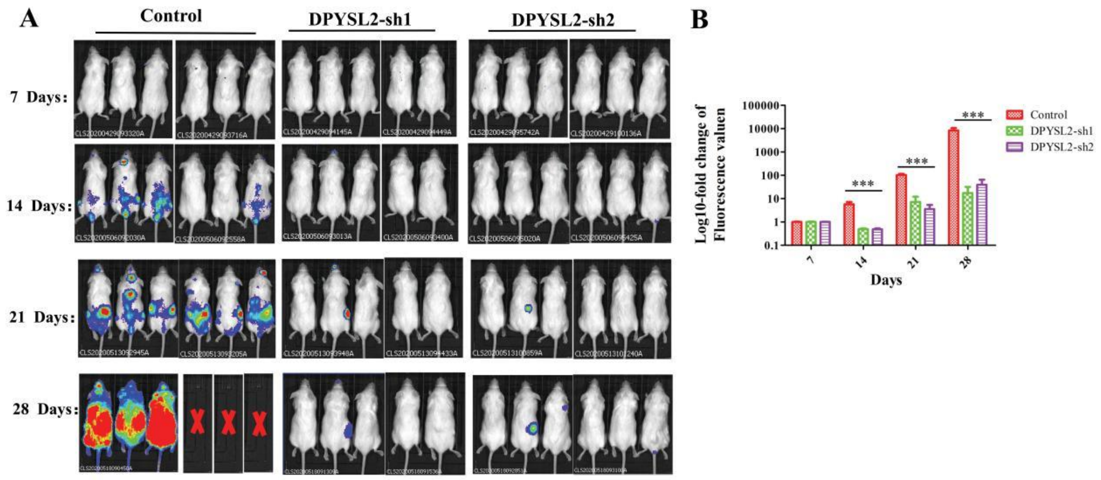

FigureS4 Tumor burden of dorsal view by in vivo imaging (A) In vivo imaging results of the back of mice on the 7th, 14th, 21th, and 28th days after cell injection. (B) Quantitative analysis results of in vivo imaging results of the back of mice. $P < 0.001$ ***

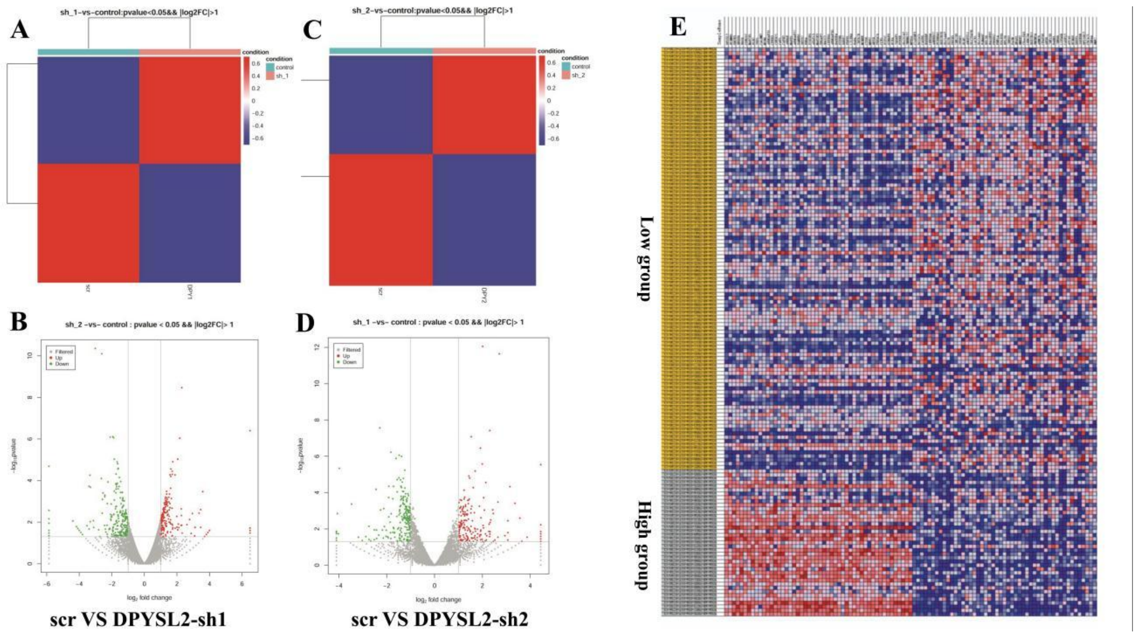

FigureS5 The gene expression profile had a significantly difference between control and DPYSL2 KD cells (A-B) Heatmap (A) and Volcano (B) showed the DEGs of "p value<0.05&& |log2FC| > 1" between MV4-11 control and MV4-11 DPYSL2-sh1 cells by RNA-seq. (C-D) Heatmap (A) and Volcano (B)showed the DEGs of "p value<0.05&& |log2FC| > 1" between MV4-11 control and MV4-11 DPYSL2-sh2 cells by RNA-seq. (E) Heatmap showed the profile of genes between the DPYSL2-high group and DPYSL2-low group from TCGA database analyzed by GSEA.

Table S1 The Antibodies used in this study

Antibody	Catalog Number	Vendor	RRID
GAPDH	5174	CST	AB_10622025
STAT3	4904	CST	AB_331269
Phospho-STAT3	9145	CST	AB_2491009
STAT5	94205	CST	AB_2737403
Phospho-STAT5	9359	CST	AB_823649
PI3K P110	4249	CST	AB_2165248
PI3K P85	4257	CST	AB_659889
AKT	2920	CST	AB_1147620
Phospho-AKT	4060	CST	AB_2315049
GSK-3b	9315	CST	AB_490890
Phospho-GSK-3b	4257	CST	AB_659889
Phospho-BCL2	2827	CST	AB_659950
Caspase3	9662	CST	AB_331439
PARP	9532	CST	AB_659884
DPYSL2	ab129082	Abcam	AB_11154701
CD14	ab28061	Abcam	AB_726117

JAK2	17670-1-AP	Protein tech	AB_2811138
BCL2	26593-1-AP	Protein tech	AB_2818996
CD11b	CD11B04	BD	AB_2536481

Table S2 Top20 pathways between DPYSL2 low group and high group from TCGA cohort by GSEA

NAME	SIZE	NES	NOM p-value	FDR q-value
HALLMARK_COMPLEMENT	200	2.0962157	0	0.011408063
HALLMARK_IL2_STAT5_SIGNALING	195	2.0682492	0	0.00739952
HALLMARK_INFLAMMATORY_RESPONSE	198	2.0354717	0.004728132	0.00777764
HALLMARK_NOTCH_SIGNALING	32	2.0272298	0.002132196	0.007165429
HALLMARK_IL6_JAK_STAT3_SIGNALING	87	1.9595119	0.002304148	0.009697801
HALLMARK_INTERFERON_GAMMA_RESPONSE	198	1.9585541	0.004192872	0.008625236
HALLMARK_APOPTOSIS	159	1.925283	0	0.011685654
HALLMARK_P53_PATHWAY	192	1.9194914	0.004246285	0.011195265
HALLMARK_TNFA_SIGNALING_VIA_NFKB	198	1.9020792	0.006696429	0.011937592
HALLMARK_TGF_BETA_SIGNALING	54	1.880274	0	0.015321779
HALLMARK_PI3K_AKT_MTOR_SIGNALING	104	1.854644	0	0.018033745
HALLMARK_INTERFERON_ALPHA_RESPONSE	95	1.8353312	0.028397566	0.0193947
HALLMARK_ALLOGRAFT_REJECTION	195	1.834353	0.00896861	0.018237963
HALLMARK_APICAL_JUNCTION	194	1.8260956	0.002336449	0.017701585
HALLMARK_HYPOXIA	191	1.8026996	0.002132196	0.020744642
HALLMARK_XENOBIOTIC_METABOLISM	197	1.7787178	0.004640371	0.024381388
HALLMARK_CHOLESTEROL_HOMEOSTASIS	73	1.7499584	0.02771855	0.03032576
HALLMARK_KRAS_SIGNALING_UP	194	1.738924	0.010443864	0.03119232
HALLMARK_MITOTIC_SPINDLE	198	1.7192645	0.010752688	0.033900883
HALLMARK_REACTIVE_OXYGEN_SPECIES_PATHWAY	47	1.6812763	0.014314928	0.043177735
